# Empowering Older Persons through Creative Engagement: A Feasibility Study of ‘The House of Evergreen Arts’ among Chinese Community Members in Newcastle, England

**DOI:** 10.1007/s10823-025-09551-y

**Published:** 2026-01-27

**Authors:** Lisa Evans, Cath Darling, Pui Lee, Lai-yee Tsang, Simon Luddington, Frank Ho-yin Lai

**Affiliations:** 1 School of Communities and Education, Northumbria University, Newcastle upon Tyne, United Kingdom; 2 Freelance Artist and Arts Educator, Newcastle Upon Tyne, United Kingdom; 3 Search – Services For Older People CIO, Newcastle Upon Tyne, United Kingdom; 4 Mental Health Research Centre, Hong Kong Polytechnic University, Hong Kong, China

## Abstract

Creative engagement through arts and crafts has been increasingly recognised as a valuable tool for promoting well-being among older persons. This exploratory study examines the feasibility and experiential impact of a five-month Older Persons Art Project designed for older members of Newcastle’s Chinese community. Participants aged 60 and above were recruited through community organisations and engaged in weekly textile-based workshops. Activities included embroidery, indigo dyeing, block printing, and observational drawing. Data were collected through written reflections and semi-structured video interviews, and analysed using Braun and Clarke’s six-phase thematic analysis framework. The authors aimed to: (1) explore how artistic activities may support cognitive engagement and fine motor coordination; (2) examine the role of social interaction in fostering emotional well-being; (3) understand the influence of cultural heritage on identity and belonging; (4) identify challenges faced by older persons in creative engagement and strategies used to address them; and (5) offer practical recommendations for future community-based arts initiatives. Thirteen participants (aged 64–80) engaged in the programme. Five key themes emerged: learning and skill development, social interaction and community, enjoyment and satisfaction, cultural heritage and identity, and challenges and perseverance. Participants described increased confidence, a sense of achievement, and strengthened cultural connection The findings suggest that arts-based programmes can offer meaningful opportunities for older persons to engage creatively, build social bonds, and reconnect with cultural identity. While the study does not claim measurable outcomes, it highlights the potential of participatory arts initiatives to support healthy ageing through inclusive and culturally responsive design.(252 words).

## Introduction

Creative engagement through the arts has long been recognised as a valuable contributor to well-being across the life course. Participation in creative pursuits such as painting, sculpture, and other artistic modalities can markedly augment both physical and psychological health. Creative expression provides a non-verbal outlet for emotional experiences, supporting emotional regulation and reducing stress (Mastandrea et al., 2019). Additionally, arts engagement stimulates cognitive processes such as memory, attention, and problem-solving, which are particularly beneficial for older persons in maintaining mental acuity and mitigating cognitive decline (Chacur et al., 2022). Arts-based programmes also foster social interaction and community building, which are essential for mental wellness in later life. Group-based creative activities can reduce isolation and promote a sense of belonging (Crealey et al., 2023). These benefits are especially relevant for older persons from minoritised cultural backgrounds, where culturally resonant art forms can support identity and intergenerational connection.

For older persons of Chinese heritage, traditional artistic practices such as calligraphy, embroidery, and indigo dyeing offer meaningful ways to engage with cultural identity and legacy. These practices can reinforce a sense of pride and continuity, contributing to psychological well-being. This involvement can boost their individual identity and a feeling of enjoyment, thus aiding in their general psychological health. Involvement in culturally significant artistic expressions assists older persons in sustaining a connection to their heritage, facilitating a sense of communal belonging. Liu et al. (2023) found that culturally tailored participatory arts programmes can empower older Chinese persons and support mental health (Liu et al., 2023).

Despite growing evidence of the benefits of arts engagement for older persons, there remains a gap in understanding how such programmes can be designed and implemented in culturally specific contexts, particularly within diaspora communities in regional UK settings. This study addresses that gap by exploring a community-based arts initiative developed in Newcastle upon Tyne for older persons of Chinese descent.

The programme under study was co-developed with local community organisations. It aimed to provide older persons with opportunities to engage in textile-based creative activities, including embroidery, indigo dyeing, and block printing, while fostering social connection and cultural expression. Rather than evaluating outcomes through pre- and post-measures, this study adopts a qualitative, exploratory approach to examine the feasibility and experiential impact of the programme. It focuses on how participants engaged with the sessions, the role of facilitation in supporting diverse needs, and the perceived benefits of participation.

Theoretical frameworks such as Erikson’s Theory of Psychosocial Development (Gross, 2020), Activity Theory (Tabet, 2016), Continuity Theory (Winstead et al., 2014), Sociocultural Theory of Creativity (Glăveanu, 2020), and Self-Determination Theory (Ryan & Deci, 2000) inform the interpretation of findings. Specifically, Erikson’s Theory of Psychosocial Development underscores the necessity of achieving a sense of integrity in advanced age through avenues of creative expression (Gross, 2020). Activity Theory posits the imperative for meaningful engagement to sustain a high quality of life (Tabet, 2016). Continuity Theory asserts that the maintenance of consistent activities is instrumental in preserving identity and self-concept (Winstead et al., 2014). The Sociocultural Theory of Creativity highlights how social and cultural environments shape the creative journey (Glăveanu, 2020), whereas Self-Determination Theory focuses on meeting psychological needs through the aspects of autonomy, competence, and connection (Ryan & Deci, 2000). Collectively, these theoretical frameworks provide an extensive basis for comprehending the positive ramifications of art projects on older persons, accentuating the essential roles of creativity, social engagement, cultural heritage, and personal development (Fioranelli et al., 2023a).

This research endeavour seeks to explore the feasibility and experiential impact of creative engagement on the psychological well-being, social interactions, and cultural identity of older persons involved in the “Older Persons Art Project.”

### Objectives

1. To explore how participation in textile-based artistic and craft activities may support cognitive engagement and fine motor coordination among older persons, based on self-reported experiences.
2. To examine the role of social interactions within the programme in fostering emotional support, connection, and a sense of community.
3. To understand how engagement with culturally significant art forms contributes to participants’ sense of identity, pride, and belonging.
4. To identify barriers to participation—such as physical, linguistic, or psychological challenges—and describe the facilitation strategies used to address these.
5. To generate practical insights and recommendations for designing inclusive, culturally responsive arts programmes for older persons in community settings.

## Methods

### Participants and Recruitment

Individuals were deemed eligible to participate in the project if they fulfilled the following criteria: they were 60 years of age or older at the time of enrolment, lived in the North East of England, and could attend weekly in-person sessions at the specified venues. Participation in the arts programme was open to all eligible individuals, regardless of whether they consented to participate in the research component. Those who declined interviews or recordings were still welcome to attend sessions.

Participants were required to be open to engaging in group-based artistic and craft activities and to give informed consent for their involvement in the research, which included data collection such as interviews, photography, and video recordings. Ethical approval for the study was obtained from the management board of Search Newcastle, and all procedures adhered to ethical guidelines for research with older persons.

Individuals were disqualified from participation in the research if they were below the age of 60, had significant cognitive or physical disabilities that would hinder meaningful involvement in group activities (as assessed informally during initial contact), or were unable to communicate in English, Cantonese, or Hakka—the primary languages utilised during the sessions. Additionally, those who opted out of being recorded or interviewed as part of the project’s documentation and evaluation process were excluded from the research component.

Recruitment commenced through a partnership with local community organisations, which promoted the project via mailing lists, newsletters, and social media channels. As the project progressed, additional participants joined through word-of-mouth referrals. The recruitment strategy was flexible and inclusive, accommodating the varying availability of older persons, including those temporarily overseas for family visits. As a result, the project adopted a drop-in and snowball sampling format, welcoming participants from the initial groups.

### Project Design and Setting

The Artist Residency spanned five months, with weekly sessions held at a community centre in Newcastle’s Chinatown. Sessions took place every week from 1:00 to 4:00 p.m. The project began with introductory “taster sessions”, which were short, informal workshops designed to introduce participants to different textile-based art forms and gauge interest.

Each weekly session typically included a combination of skill-building activities (e.g., embroidery, indigo dyeing, block printing), group discussion, and informal social interaction. The programme was designed to be responsive to participants’ interests and abilities, with the artist-facilitator adapting techniques and materials to accommodate varying levels of experience and physical mobility.

Over time, the group developed a strong sense of ownership, naming themselves “The House of Evergreen Arts.” Their collective efforts culminated in participation in the 2025 Chinese New Year Parade and a final public exhibition held on 3 March 2025 in Newcastle’s Chinatown. This event marked the conclusion of the residency and the formation of a new Chinese Elders Craft Group.

### Data Collection and Analysis

Participant feedback was collected through multiple formats, including written reflections, audio recordings, and video interviews. Interviews were conducted in a separate space during workshop sessions by a videographer and were recorded across three sessions prior to the final exhibition. Each interview followed a semi-structured format, lasting approximately 15–30 min. Interview questions were designed to elicit reflections on participants’ experiences, challenges, and perceived benefits of the programme. To reduce potential bias, participants were assured that their responses would remain confidential and that there were no right or wrong answers. The interviewer used neutral prompts and avoided leading questions. A sample of guiding questions is included in Appendix 1.

All narrative data were transcribed and subjected to thematic analysis, following Braun and Clarke’s (2006) six-phase framework (Braun & Clarke, 2006). This framework includes: (1) familiarisation with the data, (2) generating initial codes, (3) searching for themes, (4) reviewing themes, (5) defining and naming themes, and (6) producing the report. This approach facilitated the identification of recurring patterns and themes within the participants’ experiences. The analysis was led by the first author, with support from a bilingual postgraduate research assistant trained in qualitative methods and thematic analysis. The assistant held a Master’s degree in Public Health and had prior experience conducting community-based research with Chinese older persons. Their role included assisting with transcription accuracy checks, preliminary coding, and theme development discussions. Regular debriefing sessions were held to ensure consistency in coding and interpretation. Although formal inter-rater reliability statistics were not calculated due to the exploratory nature of the feasibility study, coding discrepancies were discussed and resolved collaboratively to enhance analytical rigour and trustworthiness.

Thematic analysis was primarily inductive, allowing themes to emerge from the data rather than being imposed by pre-existing theoretical constructs. While the study’s research objectives and psychosocial frameworks informed the interpretation of themes, no pre-defined codes were used at the outset. Instead, initial coding was conducted openly, and a preliminary codebook was developed collaboratively by the research team after the first round of coding. This codebook underwent two iterations, with revisions made to refine theme definitions and merge overlapping codes. A summary of the coding process and theme development is presented in Table 1. Additionally, details of the 28 initial codes and 18 refined codes are depicted in Appendix 2 – Initial Code and Description; Appendix 3 – Refined Code and Description; and Appendix 4 – Final Theme and Grouped Refined Codes.

**Table 1:** Coding process and theme development

Coding Phase	Description of Activities	Number of Codes/Themes	Codebook Revisions
Initial Familiarisation	Researchers read transcripts and interview notes to gain an overview of participant narratives.	N/A	None
Open Coding	Initial codes were generated inductively from the data without predefined categories.	28 initial codes	Preliminary codebook drafted
Codebook Refinement	Codes were reviewed, merged, and refined through team discussions.	18 refined codes	First revision completed
Theme Development	Codes were grouped into broader themes reflecting participant experiences.	5 themes	Second revision completed
Final Review	Themes were finalised and defined for reporting.	5 final themes	No further revisions

### Findings

Four participants discontinued after the initial taster sessions—three due to scheduling conflicts and one due to health-related issues. The remaining thirteen participants formed a core group that engaged consistently throughout the five-month programme. They comprised eleven women and two men, aged between 64 and 80. Most participants were based in Newcastle. While the majority were of Chinese descent, one participant of non-Chinese heritage joined due to a personal connection with Chinese culture. All participant identifiers used in this section are pseudonyms to protect confidentiality.

Thematic analysis of participant narrative feedback revealed five key themes: learning and skill development, social interaction and community, enjoyment and satisfaction, cultural heritage and identity, and challenges and perseverance, as depicted in Table 2.

**Table 2:** Patterns and themes of participants’ experiences

**Theme 1 - Learning and Skill Development**
• “I really enjoyed learning embroidery and have been keen to learn.” - YLL • “All was good and fun to learn together!!!” - SFC • “I am very happy to participate in a cultural and traditional handicraft project that has been around for hundreds or even thousands of years! We have learnt hand-made Block Printing, Embroidery, Sashiko and Fabric-dyeing.” - AL • “Hopefully, there will be another opportunity to learn more in the future.” - AL • “We will continue to learn, persevere and be patient to create works, and feel happy and satisfied.” - BF • “Although I’m just learning, Pui Lee the artist tutor and others help and advice me.” - LC • “I love that I can meet up and chat with friends while learning something new and creating nice things at these sessions.” - LC • “I have picked up some skills and techniques.” - YY • “We are actually learning different styles of stitching and embroidery techniques such as Japanese, French and British varieties too.” - SFC • “Learning tie-dye and block-printing has opened up our eyes about the in-depth techniques involved with the use of various materials and substances.” - SFC
**Theme 2 - Social Interaction and Community**
• “Being together is fun!” - HC & LWKH • “I love that I can meet up and chat with friends while learning something new and creating nice things at these sessions.” - LC • “We are learning from each other. We are helping each other. We are like a family.” - AL • “I will encourage other friends to participate, and I hope that Chinese friends can have a more fulfilling and healthier life in their later years.” - AC • “With the emergence of this class, I believe that I and other students will feel that in the process of participation, everyone has a fulfilling and energetic life in ordinary life.” - KAKH • “I can also interact and share with the group members.” - JI • “We encourage each other and love each other, thank you!” - BF
**Theme 3 - Enjoyment and Satisfaction**
• “I really enjoyed learning embroidery and have been keen to learn.” - “It has been such an enjoyable process. Thank you Pui for being patient when teaching us.” - YLL • “I love that I can meet up and chat with friends while learning something new and creating nice things at these sessions.” - LC • “I enjoyed learning these types of arts and crafts.” - YY • “I am very happy to participate in a cultural and traditional handicraft project that has been around for hundreds or even thousands of years!” - AL • “I, FW, thoroughly enjoyed this Art & Craft project and learnt many new skills.” - FW • “I find it very interesting and enjoy the process of mutual cooperation and personal effort.” - AC • “I create colourful works and feel satisfied and happy.” - JI • “I enjoy the satisfaction and adoration of a finished work by myself and others.” - JI • “I had a great time at the Chinese New Year parade and many thanks to the teacher/instructor and everyone at the embroidery class who are ever so helpful!” - AC
**Theme 4 - Cultural Heritage and Identity**
• “The project also incorporated visits to city centre museums and art galleries for inspiration, fostering connections between participants and Newcastle’s rich cultural resources.” - PL • “Additionally, the group has explored aspects of Chinese heritage and cultural identity, drawing inspiration from traditional indigo fabric-dye techniques from Yunnan province; as well as other textile decoration techniques that its neighbouring country in East Asia has to offer.” - PL • “We are in our early 80s. We used to live in Hong Kong and always lived in Hong Kong. A few years ago, we decided to live with our daughter and her family in Newcastle.” - HC & LWKH • “I am very happy to participate in a cultural and traditional handicraft project that has been around for hundreds or even thousands of years!” - AL • “I hope that Chinese friends can have a more fulfilling and healthier life in their later years.” - AC • “I wish to thank South Mountain Chinese Older People’s Association in Newcastle for the opportunity of the learning of embroidery and various artistic techniques and be proud.” - BF
**Theme 5 - Challenges and Perseverance**
• “Throughout the project, I felt the scenes were warm and jocular. Especially seeing male members struggling with needles and threads! I can’t resist to raise a smile and at times bursts of laughter when someone howled due to fingers pricked!” - SFC • “We will continue to learn, persevere and be patient to create works, and feel happy and satisfied.” - BF • “It has not been easy to embroider, dye and print but under the guidance from a caring and thoughtful teacher, we gradually understand and becoming more creative with determination.” - BF • “What matters is persistence and perseverance.” - JI

### Learning and Skill Development

Participants reported significant learning and development of new artistic skills. Many had little or no prior experience with textile arts, yet over the course of the project, they acquired a range of techniques, including hand-stitching, embroidery, appliqué, Japanese Sashiko, and Chinese indigo dyeing using fold, wrap, and resist methods. They also learned block printing using linocut and polystyrene, as well as observational drawing and smartphone photography, which they used to document their experiences during two research trips to Newcastle and Gateshead.

Several participants chose to continue practising these skills outside of the weekly sessions, often preparing additional pieces for the final exhibition. For instance, like participant JI shared, “I started everything from scratch. But it feels very interesting. I keep learning until I complete the creation… I enjoy the satisfaction and adoration of a finished work by myself and others.” Similarly, participant YLL expressed, “I really enjoyed learning embroidery and have been keen to learn. It has been such an enjoyable process.”

### Social Interaction and Community

Participants described the workshops as spaces for meaningful social connection. Friendships developed through informal conversations and shared creative tasks. The relaxed atmosphere and shared cultural references helped foster a sense of community.

As participants became more familiar with one another, they began to offer constructive feedback and mutual encouragement. Participant LC noted, “I love that I can meet up and chat with friends while learning something new and creating nice things at these sessions.” Participants also shared their work with family and friends, taking pride in their creative achievements. For example, participant AC reflected, “Even at the age of 70, I still have the opportunity to work together with a group of seniors, unleash my creativity, and complete small works… I hope that Chinese friends can have a more fulfilling and healthier life in their later years.”

### Enjoyment and Satisfaction

Participants consistently described the sessions as enjoyable and fulfilling. Many became deeply absorbed in their creative work and expressed enthusiasm for the process.

The atmosphere during workshops was generally lively and positive, with participants engaging enthusiastically in both the artistic activities and the social aspects of the sessions. Participant KAKH described the experience as “a little extraordinary …. With the emergence of this class, I believe that other students and I will feel that in the process of participation, everyone has a fulfilling and energetic life in ordinary life”. The group’s involvement in the Chinese New Year Parade was frequently mentioned as a highlight, contributing to a shared sense of achievement.

### Cultural Heritage and Identity

Engagement with traditional Chinese and Japanese textile techniques prompted reflection on cultural identity and personal history. Participants discussed the significance of indigo dyeing techniques from the Bai tribe in Yunnan Province and compared them with Japanese Shibori methods. These activities sparked conversations about sustainability, upcycling, and the meditative aspects of slow craft.

**Fig. 1:**
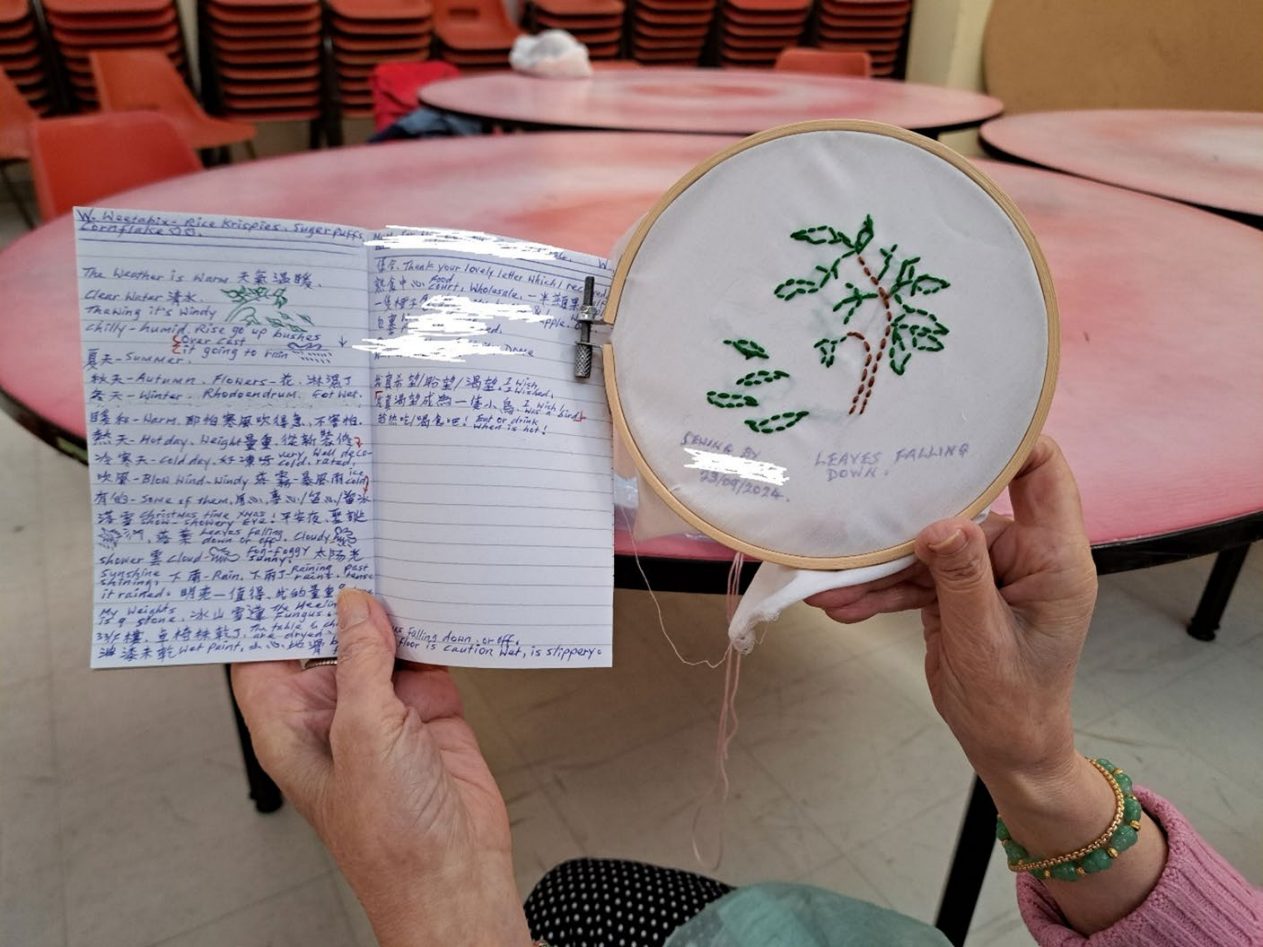
Exploration of traditional Chinese and Japanese textile techniques

**Fig. 2:**
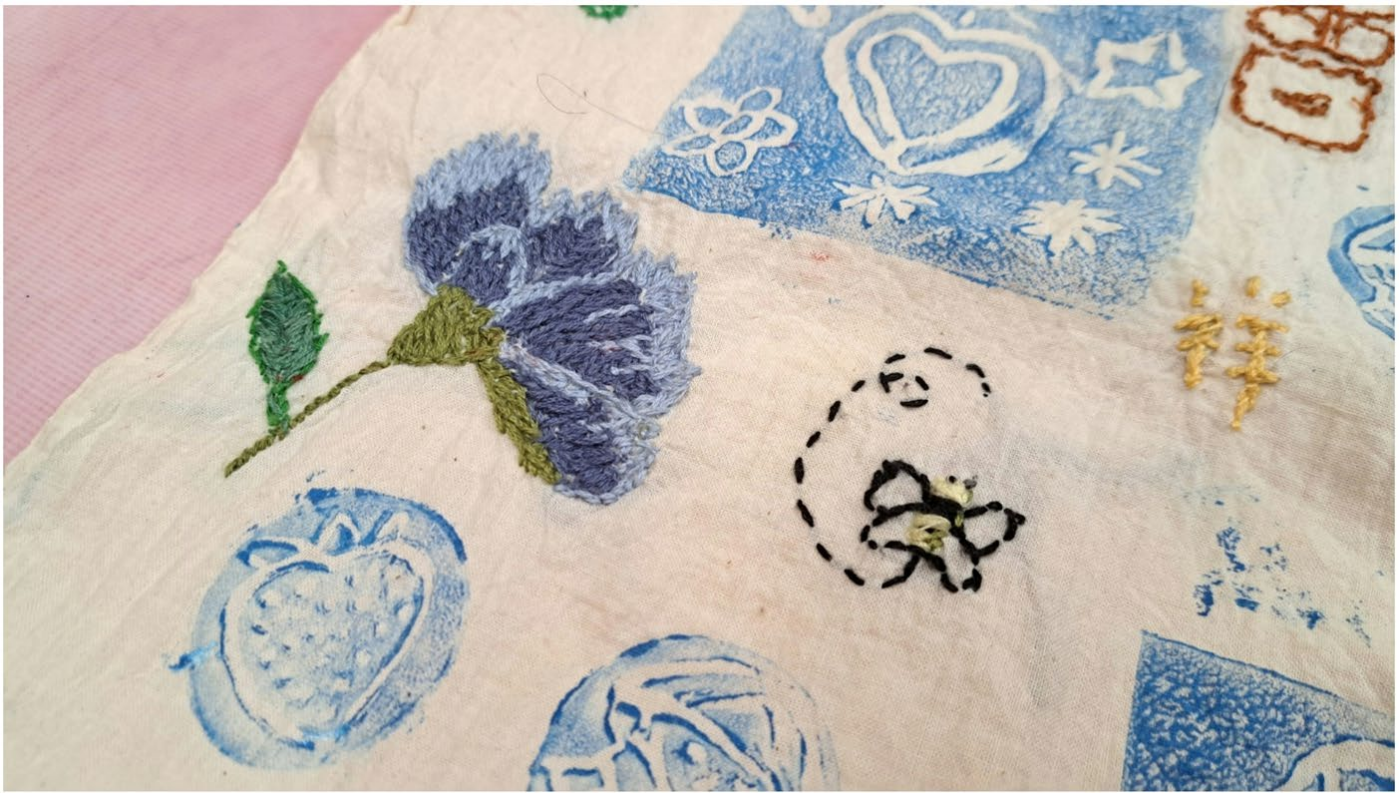
The indigo dyeing techniques

Through the exploration of traditional Chinese and Japanese textile techniques, participants were able to reconnect with their cultural roots and reflect on their personal histories, Fig. 1. The indigo dyeing techniques introduced during the project, originating from the Bai tribe in Yunnan Province, were compared with similar Japanese methods such as Shibori, Fig. 2. These activities prompted discussions about sustainability, upcycling, and the meditative aspects of slow craft. Participant AL remarked, “I am very happy to participate in a cultural and traditional handicraft project that has been around for hundreds or even thousands of years… We are learning from each other. We are helping each other. We are like a family.” For participants HC & LWKH, who had recently moved from Hong Kong, the project offered a meaningful opportunity to engage with cultural practices they had not explored before: “When we were in Hong Kong, we didn’t have the opportunity to engage in learning the arts of stitching or embroidery. Now at our age, we are so pleased to be given the opportunity.”

### Challenges and Perseverance

Participants encountered a range of challenges, including unfamiliarity with techniques, language barriers, and physical limitations such as joint pain and reduced mobility. Despite these difficulties, participants described a strong sense of perseverance and mutual support.

The artist facilitator provided tailored support, and participants frequently helped one another, creating a collaborative and inclusive environment. Participant BF reflected, “It has not been easy to embroider, dye and print, but under the guidance from a caring and thoughtful teacher, we gradually understand and become more creative with determination. Very rewarding indeed.” Participant JI echoed this sentiment, stating, “What matters is persistence and perseverance.”

These findings reflect the participants’ journey through learning, connection, and creative expression. The themes are grounded in their own narratives and illustrate the multifaceted impact of culturally relevant arts engagement in later life.

## Discussion

This study explored the feasibility and experiential impact of a community-based older persons art project on individuals’ psychological well-being, social interactions, and cultural identity through creative engagement. The findings are discussed below in relation to the five research objectives, supported by relevant theoretical frameworks and peer-reviewed literature.

### Artistic and Craft Activities: Cognitive Engagement and Motor Coordination

While this study did not include pre- and post-measures of cognitive or motor function, participants described feeling mentally stimulated and proud of acquiring new skills. Activities such as embroidery, indigo dyeing, and block printing required sustained attention and fine motor coordination, which participants referenced in their reflections. These findings align with a growing body of evidence suggesting that arts engagement can serve as a non-pharmacological intervention to support cognitive health in ageing populations (Liu et al., 2024).

These experiences align with existing literature suggesting that arts engagement may support cognitive health and manual dexterity in older persons. A recent systematic review concluded that both active and receptive arts engagement significantly reduce cognitive decline and enhance quality of life in older persons(Fioranelli et al., 2023b). Similarly, Huang et al. (2025) found that integrated social-art interventions led to short-term improvements in global cognitive function among older persons with mild cognitive impairment (Huang et al., 2025). These studies support the observed outcomes in this project, where participants reported feeling mentally stimulated (Krell-Roesch et al., 2017) and proud of mastering new skills (Moore & Campbell, 2010).

However, the findings should be interpreted as self-reported perceptions rather than objective outcomes. The embodied nature of craft practices (Bae, 2013) and the concept of “use-dependent plasticity” (Almond, 2010; Privodnova et al., 2020) offer useful theoretical lenses, but further research is needed to assess these effects systematically.

### Social Interactions to Mental Health and Well-Being

**Fig. 3:**
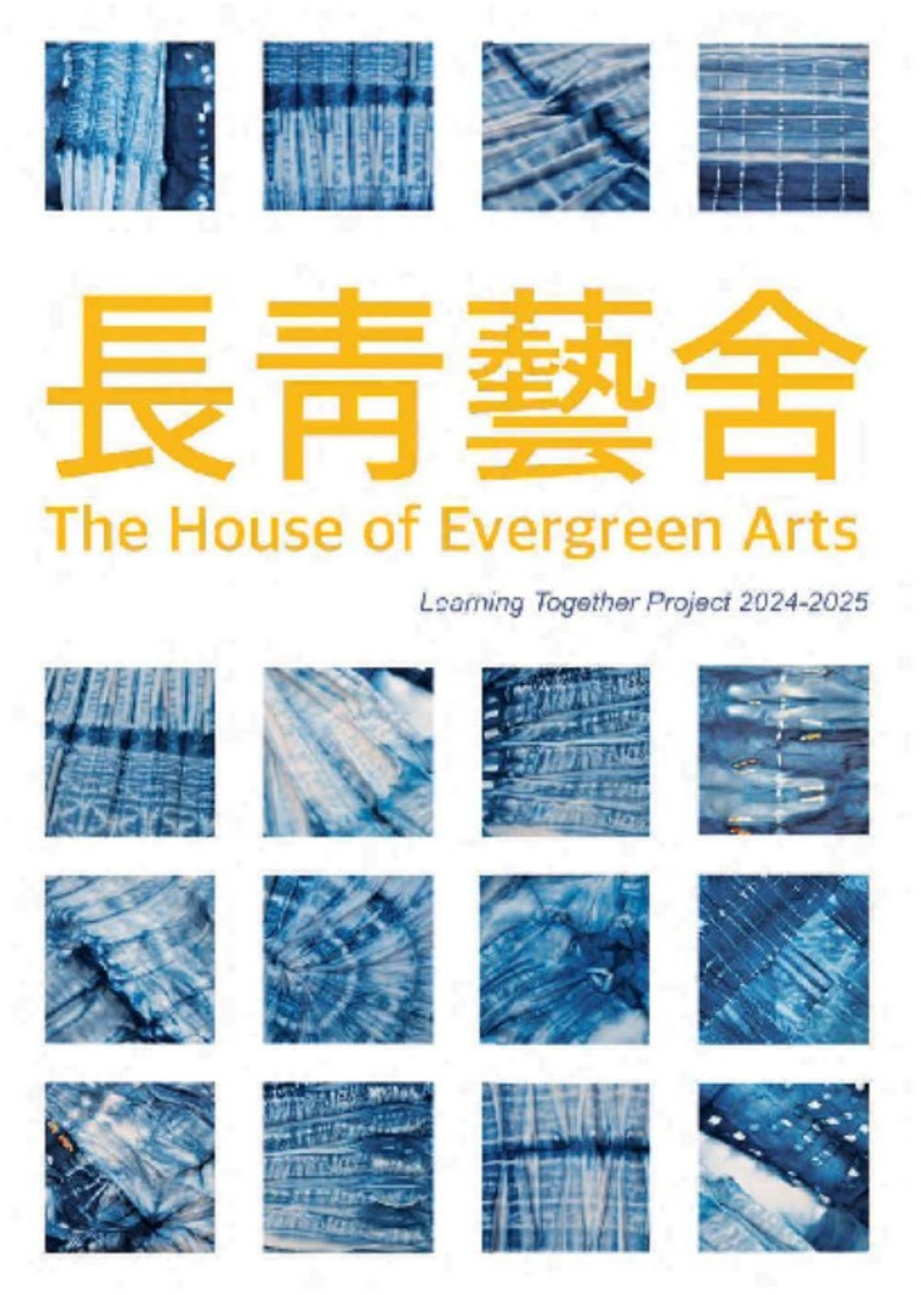
Brochure of “The House of Evergreen Arts” exhibition

Social interaction emerged as a central component of success (Li et al., 2023). Social connection emerged as a central theme in participants’ narratives. They described forming new friendships, engaging in collaborative decision-making, and supporting one another throughout the creative process (Wikström, 2002). These interactions contributed to a sense of belonging, emotional support, and shared purpose (Thomas et al., 2013). This is consistent with Self-Determination Theory’s emphasis on relatedness (Ryan & Deci, 2000) and Continuity Theory’s focus on maintaining social roles (Ng et al., 2021). The communal nature of the workshops, shared rituals, and the group’s self-naming (“The House of Evergreen Arts”), Fig. 3, fostered a collective identity and sense of purpose. These findings echo prior research showing that participatory arts programmes can enhance social well-being in older persons (Crealey et al., 2023).

### Cultural Heritage and Identity

Participants’ engagement with traditional Chinese and Japanese textile techniques provided opportunities to reconnect with cultural heritage and reflect on personal histories. This aligns with the Sociocultural Theory of Creativity, which emphasises the role of cultural context in shaping creative expression (Adams-Price & Morse, 2024). This engagement fostered a sense of pride, continuity, and identity, particularly among participants who had limited prior opportunities to express their cultural background in public or creative settings (Mackell et al., 2022). Additionally, participants described feelings of pride, continuity, and emotional resonance when engaging with heritage crafts. These reflections support previous findings that culturally relevant art activities can enhance self-esteem and intergenerational connection (Tan, 2018).

This aspect of the project aligns with the Sociocultural Theory of Creativity, which emphasises the role of cultural context in shaping creative expression. By engaging with heritage crafts, participants were not only learning new skills but also participating in a form of cultural preservation and transmission. This process can be particularly empowering for older persons, who may feel that their cultural knowledge is undervalued in contemporary society (Adams-Price & Morse, 2024).

### Challenges and Facilitation Strategies

Despite the overall success of the project, participants faced several challenges, including physical limitations (e.g., joint pain, reduced mobility), language barriers, and initial self-doubt (Liu et al., 2024; McGuire et al., 2005). These obstacles are common in arts-based interventions with older populations (Lee et al., 2025).

Facilitation played a key role in overcoming these barriers. The artist-facilitator adapted tasks to suit different ability levels and fostered an inclusive environment. The artist facilitator also provided individualised support to suit different ability levels. Peer support and collaborative learning also helped participants build confidence and persist through difficulties. These findings echo those of Huang et al. (2025), who noted that structured, professionally guided art sessions can help older persons overcome initial hesitations and engage meaningfully in creative tasks (Huang et al., 2025). Moreover, these strategies reflect best practices in inclusive arts programming and challenge stereotypes about older adults’ adaptability and creativity (Oh et al., 2025).

### Strategic Recommendations for Future Initiatives

Based on the outcomes of this project, several strategic recommendations can be made for future for future arts-based programmes targeting older persons. First, adopting a relational approach that values the shared journey of creation over the final product, as outlined in Bourriaud’s concept of *Relational Aesthetics* (Abrudan, 2023). The value of these activities lies not only in the final artistic product but also in the shared journey of creation (Liu et al., 2024).

Second, to design culturally responsive programmes that reflect participants’ heritage and lived experiences (Liu et al., 2023). Culturally responsive programming is essential for fostering meaningful engagement, especially among minoritised older persons. Programmes that incorporate familiar cultural symbols, languages, and artistic traditions can enhance emotional resonance and reduce barriers to participation. For example, Liu et al. (2023) co-designed a Chinese calligraphy-based art programme with older persons in Hong Kong, demonstrating how cultural relevance empowered participants and supported emotional wellbeing. For Chinese elders, activities that draw upon textile arts, calligraphy, or traditional festivals may evoke positive memories and affirm identity, contributing to psychological wellbeing. Previous study found that arts-based research with immigrant and racialised older persons enhanced social connectedness and self-empowerment, especially when cultural and linguistic relevance was prioritised (Salma et al., 2024). Moreover, culturally grounded approaches help counteract feelings of marginalisation and promote social inclusion, particularly in diasporic communities. Scholars emphasised the importance of collective impact and interdisciplinary collaboration in delivering culturally meaningful arts programmes for older persons in South Korea and Finland (Lee et al., 2025).

Third, to ensure accessibility and flexibility, including adaptive materials, drop-in formats, and multilingual facilitation (Chacur et al., 2022; Lee et al., 2025). This includes adapting materials and techniques to accommodate diverse physical and cognitive abilities, and offering drop-in formats or hybrid models to support varying levels of attendance. Fourth, to invest in skilled facilitation. Artist-facilitators should be both technically proficient and sensitive to the needs of older persons (Liu et al., 2023). Finally, to use mixed-methods evaluation to capture both qualitative insights and, where appropriate, quantitative outcomes. These recommendations are intended to guide future feasibility studies and programme development, rather than to generalise outcomes beyond the scope of this exploratory research.

### Limitations

This study has several limitations that should be considered when interpreting the findings.

First, the study adopted a qualitative, exploratory design without pre- and post-programme measures of cognitive, motor, or psychosocial outcomes. As such, the findings reflect participants’ subjective experiences and perceptions rather than objectively assessed changes. The reliance on self-reported data introduces potential biases, including recall bias (Brusco & Watts, 2015; Dalziel et al., 2018) and social desirability bias (Bergen & Labonté, 2020; Bispo Júnior, 2022).

Second, the sample size was relatively small (*n* = 13), and participants were recruited through community organisations and word-of-mouth referrals. This may have resulted in selection bias, as individuals who chose to participate may have been more socially motivated or predisposed to benefit from arts engagement than the general population of older persons.

Third, the demographic scope of the sample—primarily older persons of Chinese descent living in Newcastle—limits the generalisability of the findings to other cultural or geographic contexts. Future studies should aim to include more diverse participants across socio-economic backgrounds and regions.

Fourth, while observational insights were noted during the programme, they were not systematically collected or analysed as part of the study design. This limits the ability to triangulate findings across different data sources.

Fifth, the availability of resources—including materials, venue access, and qualified facilitators—may pose challenges for replicating similar programmes elsewhere. Securing adequate financial support and infrastructure is essential for sustainability.

Finally, the study did not assess long-term outcomes. Future research should consider longitudinal designs to explore the enduring impact of arts engagement on well-being, skill retention, and community participation. Additionally, future initiatives should prioritise inclusive design, ensuring accessibility for individuals with physical, cognitive, or linguistic challenges.

## Conclusion

This exploratory study has highlighted the value of engaging older persons in culturally relevant, community-based artistic and craft activities. Through qualitative analysis of participant narratives, the project revealed meaningful experiences of learning, connection, and cultural expression.

Rather than measuring outcomes such as cognitive stimulation or motor coordination directly, the study captured participants’ reflections on acquiring new skills and feeling mentally engaged. These insights suggest that creative engagement may support aspects of healthy ageing, though further research is needed to assess these effects systematically.

Equally important were the social benefits described by participants. They formed new relationships, shared experiences, and developed a strong sense of community. The workshops provided structure and emotional support, contributing to a sense of belonging and purpose. These findings underscore the importance of relational and participatory approaches in designing programmes for older populations.

The integration of cultural heritage into the creative process enriched the experience. Participants reconnected with traditional Chinese and East Asian textile practices, fostering a renewed sense of identity and pride. This cultural engagement supported personal reflection and offered opportunities for intergenerational and intercultural dialogue.

Despite initial challenges—including physical limitations, language barriers, and unfamiliarity with techniques—participants demonstrated resilience and adaptability. Facilitation strategies and peer support were key to creating an inclusive and empowering environment.

In conclusion, the Older Persons Art Project offers a promising model for creative ageing, demonstrating how arts-based programmes can foster personal growth, social connection, and cultural affirmation. Future initiatives should build on these insights by embedding arts engagement into community health and wellbeing strategies, ensuring that older persons have continued access to enriching and culturally meaningful experiences.

## Data Availability

The data that support the findings of this study are available from the corresponding author upon reasonable request.

## Appendix 1 - Guiding Questions for Participant Interviews

**1. Experience and Engagement**

- Can you describe your experience participating in the art workshops?
- What kinds of activities did you enjoy the most? Why?
- Were there any techniques or skills that were new to you?

**2. Personal Impact**

- How did participating in the sessions make you feel?
- Did you notice any changes in your confidence or motivation during the project?
- Have you continued any of the creative activities outside of the sessions?

**3. Social Interaction**

- Did you get to know new people through the workshops?
- How would you describe the atmosphere of the group?
- What role did social interaction play in your experience?

**4. Cultural Connection**

- Did the activities help you connect with your cultural heritage in any way?
- Were there any memories or traditions that came to mind during the sessions?
- How important was it for you to explore traditional techniques like indigo dyeing or embroidery?

**5. Challenges and Support**

- Were there any parts of the project that you found difficult?
- How did you overcome those challenges?
- Was there anything that helped you feel supported during the sessions?

**6. Reflections and Recommendations**

- What did you take away from the experience?
- Would you recommend this kind of programme to others?
- What suggestions would you make for future workshops like this?

## Appendix 2

**Table 3:** Initial code and description

Initial Code	Description
• Sense of Belonging • Cultural Expression • Learning New Skills • Memory Recall • Social Interaction • Language Use • Physical Engagement • Emotional Release • Confidence Building • Routine Establishment • Intergenerational Sharing • Artistic Enjoyment • Health Reflection • Community Visibility • Identity Exploration • Supportive Environment • Language Barriers • Mobility Challenges • Sensory Experience • Group Cohesion • Facilitator Role • Feedback Reception • Creative Freedom • Time Constraints • Personal Growth • Civic Pride • Language Learning • Art as Therapy	• Feeling part of a group or community • Opportunity to express Chinese heritage • Acquiring artistic techniques • Triggering personal memories • Engaging with peers • Using Cantonese, Hakka, or English • Using hands and body in art-making • Expressing emotions through art • Gaining self-assurance • Creating a weekly structure • Talking about family and youth • Pleasure from creative activities • Discussing physical or mental health • Being seen in public events • Reflecting on personal identity • Feeling safe and encouraged • Challenges in communication • Physical limitations • Textures, colours, and materials • Bonding with others • Impact of artist-facilitator • Responding to others’ comments • Autonomy in artistic choices • Limited time for activities • Development over time • Pride in public exhibition • Improving English or Chinese • Healing through creativity

## Appendix 3

**Table 4:** Refined code and description

Refined Codes	Description
• Community Connection • Cultural Identity • Skill Development • Emotional Wellbeing • Physical and Cognitive Engagement • Social Support • Memory and Reflection • Creative Autonomy • Facilitation Impact • Routine and Structure • Personal Transformation • Civic Engagement • Communication Challenges • Language Practice • Enjoyment • Physical Challenges • Cultural Engagement • Learning Process	• Sense of Belonging, Group Cohesion, Community Visibility • Cultural Expression, Identity Exploration • Learning New Skills, Language Learning • Emotional Release, Art as Therapy, Confidence Building • Physical Engagement, Mobility Challenges, Sensory Experience • Social Interaction, Supportive Environment • Memory Recall, Health Reflection, Intergenerational Sharing • Creative Freedom, Artistic Enjoyment • Facilitator Role, Feedback Reception • Routine Establishment, Time Constraints • Personal Growth • Civic Pride • Language Barriers • Language Use • Artistic Enjoyment • Mobility Challenges, Time Constraints • Cultural Expression, Traditional Festivals • Learning New Skills, Feedback Reception

## Appendix 4

**Table 5:** Final theme and grouped refined codes

Final Theme	Grouped Refined Codes
Learning and Skill Development Social Interaction and Community Enjoyment and Satisfaction Cultural Heritage and Identity Challenges and Perseverance	Skill Development, Physical and Cognitive Engagement, Memory and Reflection, Personal Transformation, Language Practice, Learning Process Community Connection, Social Support Emotional Wellbeing, Creative Autonomy, Enjoyment Cultural Identity, Civic Engagement, Cultural Engagement Facilitation Impact, Routine and Structure, Communication Challenges, Physical Challenge
